# INCIDENT AND RECURRENT FALLS AMONG NON-HISPANIC BLACK AND HISPANIC MEN WITH CHRONIC CONDITIONS

**DOI:** 10.1093/geroni/igad104.2562

**Published:** 2023-12-21

**Authors:** Temitope Olokunlade, Ledric Sherman, Mark Benden, Gang Han, Matthew Smith

**Affiliations:** Texas A&M University, College Station, Texas, United States; Texas A&M University School of Public Health, College Station, Texas, United States; Texas A&M University School of Public Health, College Station, Texas, United States; Texas A&M University, College Station, Texas, United States; Texas A&M University, College Station, Texas, United States

## Abstract

While much is known about the complexities of fall-related risks among older adults, less is known about the risk for falls among men, and especially older non-Hispanic Black and Hispanic men with chronic conditions. This study examined factors associated with incident falls (1 fall) and recurrent falling (2+ falls) among non-Hispanic Black and Hispanic men ages ≥60 years with ≥1 chronic condition. Collected with a web-delivered questionnaire, data were analyzed from a national sample of 779 non-Hispanic Black (58.8%) and Hispanic (41.2%) men. To assess incident and recurrent falls, the number of self-reported falls in the past year was trichotomized (0 falls vs. 1 fall vs. 2+ falls) and used as the dependent variable. A multinomial logistic regression was fitted to assess factors associated with incident and recurrent falls. The model adjusted for sociodemographics, disease characteristics, health status, and social support. On average, participants were age 66.8 (±5.4) years and reported 3.8 (±2.7) chronic conditions. Seventy-three percent of men reported 0 falls, 12.6% reported 1 fall, and 14.4% reported 2+ falls in the past year. Relative to men reporting 0 falls, Hispanic men (P< 0.05), men with worse general health status (P< 0.05), and those with clinical depression (P< 0.05) were more likely to report incident and recurrent falls, respectively. Men with more comorbidities (P< 0.05) and those with less help/support to manage health problems (P< 0.05) were more likely to report recurrent falling. Findings highlight the importance of multi-component interventions to prevent falls by strengthening disease self-management, addressing mental health, and introducing social supports.

